# Mill and Mental Phenomena: Critical Contributions to a Science of Cognition

**DOI:** 10.3390/bs3020217

**Published:** 2013-04-22

**Authors:** Steven L. Bistricky

**Affiliations:** Department of Clinical Health and Applied Sciences, University of Houston-Clear Lake, 2700 Bay Area Boulevard, Houston, TX 77058, USA; E-Mail: bistricky@uhcl.edu; Tel.: +1-281-283-3439

**Keywords:** John Stuart Mill, attention, meta-cognition, cognitive neuroscience, cognitive therapy

## Abstract

Attempts to define cognition preceded John Stuart Mill’s life and continue to this day. John Stuart Mill envisioned a science of mental phenomena informed by associationism, empirical introspection, and neurophysiology, and he advanced specific ideas that still influence modern conceptions of cognition. The present article briefly reviews Mill’s personal history and the times in which he lived, and it traces the evolution of ideas that have run through him to contemporary cognitive concepts. The article also highlights contemporary problems in defining cognition and supports specific criteria regarding what constitutes cognition.

## 1. Introduction

Although John Stuart Mill’s influence on economic theory and moral philosophy are well known, his contributions to the fledgling science of psychology and their far-reaching effects on the contemporary understanding of cognition are under-recognized. Mill boldly asserted that certain laws of order governed mental phenomena, and that psychology was well-suited to study these phenomena empirically and in parallel with physiology. Mill’s ideas did not arise in a vacuum, however; he reiterated certain ideas of his predecessors and contemporaries in philosophy and in the sciences. At the same time, Mill offered a new synthesis that symbolized the “mental chemistry” he would describe. As will be discussed, important aspects of Mill’s conceptions of mental phenomena have been adopted and transmitted in the marketplace of ideas across time.

This article reviews John Stuart Mill’s proposal that mental phenomena could be effectively studied through empirical means and highlights important interrelations between Mill’s ideas and modern conceptions of cognition. But an analysis tracking historical influences on current knowledge of cognition quickly encounters a core problem in cognitive science: there is no generally agreed upon answer to the question, “what is cognition?” This problem is, of course, the central topic of this Special Issue*.* Although this article cannot resolve this problem unilaterally, it attempts to identify ways that cognition is currently understood and briefly review some proposed definitional criteria for what constitutes cognition. This characterization of cognition, though provisional, must be established in order to understand and appreciate some of the influential historical ideas that have shaped modern views. After all, how can we recognize Mill’s contributions to a science of cognition if we lack a decent idea of what cognition is? 

### 1.1. Method and Contents

This is a selective review, neither stringently systematic nor exhaustive. Relevant English language texts were collected through online databases and search engines, including PsycINFO, Pubmed, and Google Scholar. Searches utilized terms such as, *cognition, cognitive, mental processes, thought, thinking*, and appropriate name combinations of *John Stuart Mill*. Relevant texts were also identified from lists of references in articles and chapters already obtained. Although Mill’s proposed “States of Mind” included thought, emotion, volition, and sensation [1], this article focuses most on thought. However, the other three “states” receive attention where a principle or the content itself is relevant to understanding Mill or cognition.

The article first introduces current issues regarding the problem of defining cognition. Then a basic framework, derived from contemporary cognitive science debate, is forwarded. Following this, the sociocultural backdrop and personal history pertaining to Mill’s theory of psychology are discussed. After this, aspects of Mill’s theory are examined in terms of how they have spread and changed over time. For example, his empiricism, associationism, and introspectionism have evolved into several contemporary concepts and methods that will be discussed. The article concludes by discussing constraints of retrospective analyses and by placing Mill’s intellectual legacy within the broader, ongoing quest to define cognition. Thus, the objective of this article is to examine Mill’s ideas about cognition and to analyze how some of those ideas have passed down into current conceptions of cognition. The present article provides evidence that specific characteristics of Mill, his society, and his ideas helped disseminate a perspective that still influences how we conceptualize cognition. 

### 1.2. What is Cognition?

Attempts to define cognition preceded John Stuart Mill’s life and, to this day, a lively debate works toward an elusive canonical definition. Common usage of *cognition* can be traced back to the 15^th^ Century when it was defined as “a getting to know, acquaintance, notion, knowledge…” [2,3]. The more recent developing science of cognition has largely defined cognitive processes in terms of tasks (e.g., perceiving a stimulus). This is not abnormal in a relatively new science. However, for the science to continue to advance, there must be an acceptable answer to the question of what makes a task cognitive in the first place. For decades, the study of cognition has largely proceeded with an implicit ethic of “we can’t really define it but we know it when we see it”. There are at least two major problems with this response. First, it encourages a relativistic notion of cognition that is highly susceptible to changes across time and academic disciplines, which would defy parsimony and encumber scientific advancement. Second, while nearly all agree that cases like perception, memory, reasoning, and concept formation fit in the category of cognition, there is significant disagreement over whether border cases, such as emotion, comprehension, and instinct fit [4]. 

In the last decade, the urgency to define *what* cognition is has increased, ironically, as cognitive science has tried to stipulate *where* cognition can occur. One position, called *extended cognition,* suggests that cognition can occur outside the brain and possibly outside of an organism. For example, extended cognition might suggest that a grocery list constitutes cognition because it serves the same function as the neural memory stores one would otherwise use to remember what to buy. In contrast, the *intracranialist* position proposes that cognition must occur inside the brain. This position would suggest that a grocery list is merely a stimulus of cognition. A key takeaway from this debate has been that the field probably cannot know *where* cognition occurs until it defines *what* it is [4]. Essential criteria, drawn from scientific practices or philosophically-grounded conditions, are needed to define cognition.

Theorists have suggested that a familiar set of attributes may often characterize cognition, although some of these attributes may not be necessary or sufficient to distinguish a phenomenon as cognitive. For example, many would agree that cognition must involve information processing in the form of computations and symbolic representations [5]. However, information processing occurs in cases in which few would call it cognition (e.g., homeostatic regulation based on temperature, blood sugar,*etc.*). So although information processing helps define cognition, it is not sufficient to distinguish it. Additionally, many would propose that a phenomenon needs to cross a certain threshold of abstraction or sophistication to qualify as cognition [6]. This position might suggest that a single neuron firing does not rise to the level of cognition. However, although an abstraction criterion may provide some characterization of cognition, it is vague, dimensional, and once again relies on case-by-case consensus building.

What might be some necessary conditions to differentiate cognition? Adams has endorsed several [7]. First, cognitive states represent and carry semantic values that can be assessed for correctness, as in true or false. In this way, the symbolic content comprising a cognitive state can misrepresent objective reality (e.g., an inaccurate belief). By contrast, a *natural sign* such as rustling tree leaves cannot misrepresent a gust of wind. It matters that cognitive states can be evaluated because mental contents, such as perceptions, concepts, and beliefs (but not sensations) thus become separable from their immediate environmental cues and causes. So a person need not be in the midst of wind to consider the concept of wind. A second important distinction is that cognition involves states composed of an internal symbol currency that *originates* within a system, carries meaning for the system, and functions for the system. This “non-derived content” is not dependent on other minds. By this criterion, current computers do not engage in cognition. A third proposed distinction is that systems that engage in cognition can operate in the service of mental contents that constitute cognition. For example, a salesperson might memorize information about prospective clients in order to pursue goal concepts of persuasiveness and sales. 

Drawing from the previously mentioned criteria this article proposes that the essence of cognition is acquiring, transforming, and utilizing information structures in direct or indirect service of goals or drives, such as optimized life regulation. Cognition interacts with, but is not reducible to, sensorimotor functions or smaller-scale phenomena, such as individual neuron activity. This description would include both conscious and unconscious processing in domains such as attention and implicit memory—where the informational contents can be brought forth for subsequent transformative mental processes. (For example, unconscious attention to subtle anger expressed in another person’s face might facilitate decision making to avoid the person. Or, as in *semantic priming*, unconsciously processing the concept of “conflict resolution” might make more accessible a collection of knowledge and skills that could be used to problem solve a tenuous interpersonal situation.) This definition of cognition, as any other existing one, would not achieve consensus, but it does reflect modern conceptions of cognition.

Of course, this article’s primary focus is on *Mill’s* 19^th^ Century ideas about cognition and their influence. To better understand his ideas, it is important to consider the sociocultural context in which Mill lived, in essence, to review some of the ideas to which Mill would have been exposed.

## 2. Sociocultural Context

John Stuart Mill was born in London in 1807, the son of esteemed philosopher, James Mill, who was an adherent to associationism and to the utilitarian philosophy of Jeremy Bentham. Associationism held that basic mental states interconnect with subsequent states to form complex memory structures and facilitate higher order mental processes. In line with this, John Stuart Mill’s comprehensive education was designed to construct a genius intellect. His rigorous schooling separated him from the imperfect influence of peers and drove him to acquire myriad mental associations. In addition to learning classic Greek texts in their native language, Mill was immersed in the British empiricism of John Locke, George Berkeley and David Hume. Also, although early 19^th^ century thinkers, including Mill, publicly distanced themselves from Gall’s phrenology of the late 18^th^ century, it was clear that many were still influenced by aspects of it [8]. For example, phrenology parted with mind-body dualism, and suggested that the brain was the *organ of the soul*, made up of various parts that employ different cognitive and emotional functions. As will be discussed, Mill likewise rejected Cartesian dualism. 

Mill grew up in the world’s most powerful nation during a period in which significant changes were occurring in the applied sciences and humanities. It was a time of innovation, exploration, and significant optimism [9]. Mill’s 1843 *A System of Logic* was distributed in an era in which slavery was abolished in Britain, formidable women’s rights movements arose, and the *Communist Manifesto* was released. Meanwhile, the population was becoming increasingly literate and educated. Overall, the times were receptive to skeptical questioning of existing hierarchical traditions on rational grounds and conceiving of and implementing new societal and intellectual order. In short, it was a competitive marketplace for ideas.

In his early twenties, Mill experienced a period of depression in which he perceived a pressing need to become independent of his father and develop a new sense of meaning. It has been suggested that Mill felt ashamed for never directly confronting his father with his differing philosophical views, and that he had felt obligated to “pay lip service to views he did not really share” [10]. In his autobiography, Mill writes that during this period, “the whole foundation on which my life was constructed fell down…I seemed to have nothing left to live for” [11].

Mill would later acknowledge his father’s imperfect but important role in preparing him, and would retain a strong sense of value for education; however he was now compelled to create new order in his philosophy. This new perspective was influenced by his contemporaries but included significant modifications as well. Mill would adopt the perspective of French philosopher, Auguste Comte, who believed that social change advances through “critical periods” in which traditional institutions were ousted and succeeded by “organic periods” where new orders are established and refined [12]. Thus, Mill disseminated an idea apt to his own life and times, an apparent ancestor to Thomas Kuhn’s thesis in *The Nature of Scientific Revolutions* [13]. Similar to Mill, Kuhn suggested that frameworks of thought do not evolve in a slow, linear fashion but rather change dramatically, resulting in new ways of understanding. Particularly relevant to this article, Kuhn stressed that a “paradigm shift” results when a new framework is adopted by a critical mass of scientific thought leaders.

More important to Mill’s life, Mill emerged from his depression determined to help usher Britain into a new organic period. Mill now eschewed the predictable, ineffective competition between entrenched liberal and conservative orders in favor of collaborative rational conservation and reform. Similarly, he reconciled religious and scientific practice through his active involvement in the Unitarian Church, which reinforced his search for meaning through scholarship and reason. Mill’s capacity for intellectual diplomacy garnered him respect. Most relevant to the present analysis, this skill set helped him chart a course for a novel discipline of psychology, which considered but differentiated itself from entrenched physiology and metaphysics. Charting this course would prove challenging, as other thought leaders believed that mental science was untenable or unimportant [14].

## 3. Mill’s Vision for Psychology

### 3.1. Empirical Science of Mind

In *A System of Logic*, John Stuart Mill asserts that psychology is a topic worthy of scientific study. He builds a strong case for the use of empiricism as a means of accumulating knowledge about causes, effects, and the need to control environmental factors in order to isolate experimental factors of interest. Mill writes, “We must, in short, follow the Baconian principle of varying the circumstances” [1]. Although Mill recognized the value of naturalistic observation, he emphasized the advantages of experimentation:

(Experimentation)…not only enables us to produce a much greater number of variations in the circumstances than nature spontaneously offers, but, also, in thousands of cases, to produce the precise sort of variation which we are in want of for discovering the law of the phenomenon—a service which nature, being constructed on a quite different scheme from that of facilitating our studies, is seldom so friendly as to bestow upon us…When we can produce a phenomenon artificially, we can take it, as it were, home with us, and observe it in the midst of the circumstances with which in all other respects we are accurately acquainted.

[1]

Mill’s primary purpose was to advocate for an empirical science of psychology. At the same time, he sought to discredit the concept of a disembodied mind championed in metaphysical philosophies. His rejection of Cartesian dualism, echoed later in cognitive psychology [15,16], is articulated in *A System of Logic*:

In the case of sensations, another distinction has also to be kept in view, which is often confounded, and never without mischievous consequences. This is the distinction between the sensation itself, and the state of the bodily organs which precedes the sensation, and which constitutes the physical agency by which it is produced. One of the sources of confusion on this subject is the division commonly made of feelings into Bodily and Mental. Philosophically speaking, there is no foundation at all for this distinction: even sensations are states of the sentient mind, not states of the body.

[1]

Lest the previous passage be thought to deny the existence of peripheral sensory-gathering hardware, Mill later clarifies:

Every sensation has for its proximate cause some affectation of the portion of our frame called the nervous system, whether this affectation originate in the action of some external object, or in some pathological condition of the nervous organization itself”.

[1]

The previous two passages are important for at least three reasons. First, they integrate what was becoming known about the peripheral nervous system in physiological circles. These notions opened the door for the study of lawful relationships between stimuli and sensory reactivity, as in the work of Ernst Heinrich Weber and Gustav Fechner. Second, these passages suggest that one’s phenomenal experience can be independently influenced by external reality *and* by internal states (*i.e.*, conditions occurring within the nervous system). Modern cognitive models of anxiety disorders incorporate similar notions, implicating a pathological bi-directional escalation of threat perception and physiological arousal [17]. Third, these two Mill passages appear to be precursors to the influential ideas of Antonio Damasio, who provides evidence suggesting the inseparable fusion of mind and body, and of reason and emotion. In his “Somatic Marker Hypothesis” Damasio has proposed that the nervous system throughout the body continually exchanges information with the brain, so that moment-by-moment humans can react adaptively to environmental circumstances or internal states [8,18]. It bears noting that Mill believed sensations were caused only by physical conditions, but he believed that thought could be caused by other states of mind (e.g., emotion, volition, sensation, or thought).

Mill was motivated to decompose mental phenomena down to the smallest constituent parts. He avoided being pigeon-holed into a reductionistic, physiological camp by way of his “mental chemistry” metaphor [19]. Mill assimilated into his psychology the chemistry phenomenon of elements fusing to become something greater than their parts, such as hydrogen and oxygen becoming water (a similar concept later emerged within the Gestalt school of cognitive psychology, referring to human perception.) Mill sensed that the study of mental states could potentially be usurped by physiology, so he sought to carve out and claim a scientific space between physiology and philosophy of mind. In *A System of Logic*, Mill acknowledges the possibility that thought, affect, and motivation may be produced by material “organs” (e.g., brain), as physiologists of his day believed [1]. He even goes so far as to say that if such causal connections became well understood, “Mental science would be a mere branch, though the highest and most recondite branch, of the science of Physiology” [1]. However, in his time Mill presented two key reasons to pursue a *psychology* of mental states. His first assertion is that (in his time) the investigative tools needed to adequately study the differences in states of the nervous system do not exist. He writes:

…that every mental state has a nervous state for its immediate antecedent and proximate cause, though extremely probable, cannot hitherto be said to be proved, in the conclusive manner in which this can be proved of sensations; and even were it certain, yet everyone must admit that we are wholly ignorant of the characteristics of these nervous states; we know not, and at the present have no means of knowing what respect one of them differs from another; and *our only mode of studying their succession and co-existences must be by observing the successions and co-existences of the mental states* of which they are supposed to be the generators or causes.”.

([1], italics added)

Mill was prescient, however, and he allowed for the possible emergence of modern technological approaches. From a modern perspective, his statement seems to identify the need for new technologies, such as functional neuroimaging and other psychophysiological approaches. In his time, this idea lay dormant. But rather than stopping with what was lacking, Mill’s second assertion builds a positive case for a psychological science of cognition, laying out a parallel relationship with physiology, where the same mental phenomena could be studied at different levels. He writes:

The successions, therefore, which obtain among mental phenomena do not admit of being deduced from the physiological laws of our nervous organization; and all real knowledge of them must continue, for a long time at least, if not always, to be sought in direct study, by observation and experiment, *of the mental successions themselves*.

[1]

This philosophy had far reaching pragmatic and theoretical implications on the study of cognition. Pragmatically, it provides a rationale for using self-report or indirect behavioral indicators of cognition as research measures. Theoretically, Mill believed mental “successions” are carried out in an associative, non-random manner. That is, mental states are connected in meaningful ways, and therefore cognition should flow in systematic and predictable directions.

### 3.2. Frontiers of Associationism

Like those who formulated and re-formulated associationism before him (e.g., Aristotle, David Hartley, James Mill, Jeremy Bentham), J.S. Mill believed that connections between mental states and rudimentary memory elements facilitate higher order cognition. For Mill, this basic associationist concept translated into applied utilitarian ideas for the betterment of individuals and society. For example, he believed that controlling the environment was the key to education and learning. As the study of psychology emerged, psychologists such as William James, Alfred Binet, Ivan Pavlov, and John Watson advanced Mill’s thinking on associationism as a science [9]. Wilhelm Wundt, commonly known as the father of modern psychology, quoted Mill and acknowledged Mill’s influence [20].

Associationism would become the forerunner to the theory of classical conditioning, in which an organism creates a memory link between stimuli that are paired during presentation. Mill’s associationism can be linked even further to modern connectionism and parallel distributed processing (PDP) models [21]. In connectionist models, sophisticated effects (e.g., mental and behavioral phenomena) emerge from activity that spreads across a network of simple units, each of which holds a differing probability of activation that is continually tuned by environmental input. Mill’s associationism also allowed for parallelism with processes that are instantiated in neural tissue. At the same time, Mill’s particular viewpoint accommodates differing cognitive functions (“faculties”). As such, it foreshadows the need within cognitive science to reconcile connectionism and modular perspectives, which suggest that information processing systems are organized into specialized components, or modules, dedicated to particular processing functions (e.g., language reception) [22]. For example, he writes:

To establish a relation between mental functions and cerebral conformations, requires not only a parallel system of observations applied to each, but…an analysis of the mental faculties…conducted without any reference to the physical conditions, since the proof of the theory would lie in the correspondence between the division of the brain into organs and that of the mind into faculties, each shown by separate evidence. To accomplish this analysis requires direct psychological study carried to a high pitch of perfection.

[23]

Mill’s ideas were rational, but the technology of the day could not provide the compelling empirical evidence that modern neuroimagining and neural network modeling have [24]. Mill knew that trying to understand the constituents and processes of cognition would be a complex task. For example, he writes, “We must decompose each chaos into single facts. We must learn to see in the chaotic antecedent a multitude of distinct antecedents, in the chaotic consequent a multitude of distinct consequents” [1]. This prescient idea has been inherited and drives theories and methods of modern cognitive neuroscience, such as the event-related potentials (ERP) technique. ERP studies present stimuli (simplified analogues to naturalistic stimuli) and elicit distinct cognitive processing (e.g., a decision whether a stimulus fits within a certain category) and concurrent psychophysiological markers of processing (e.g., P3 ERP component), which represent myriad synchronized neuronal action potentials. However, many in Mill’s day believed there would never be valid instruments to study the complexity of mental phenomena [25].

### 3.3. Capacities and Limits of Introspection

John Stuart Mill believed that psychological science could be informed by *introspection,* the practice of examining one’s own mental contents and processes. Mill’s position on introspection was stridently opposed by August Comte. Through their discourse, and from observations, Mill identified areas of cognition that would later be described and studied, such as divided attention, selective attention, and meta-cognition. Each of these areas is discussed in subsequent sections. However, Mill’s proposal about introspection first had to survive all-or-nothing assaults from Comte, such as these:

The observing and the observed organ are here the same, and its action cannot be pure and natural… In order to observe, your intellect must pause from activity; yet it is this very activity that you want to observe. If you cannot effect the pause, you cannot observe; if you do effect it, there is nothing to observe.

[14]

In response to Comte’s absolutist claim, Mill writes that introspective observation “forms a special difficulty…but a difficulty is not an impossibility”. He further alludes to “proof that the mind can not only be conscious of, but attend to, more than one, and even a considerable number of impressions at once” [23]. Modern computer modeling in cognitive science provides support to Mill’s more nuanced position, suggesting that a mind can partition its focus to observe the flow of mental contents and subordinate mental processes [26]. Mill also proposed that observation of thought need not be absolutely simultaneous to provide useful data, given human capacity for short-term memory, which keeps mental representations of recent events accessible for examination or other uses. About this form of *retrospection* [27]*,* he writes:

…it might have occurred to M. Comte that a fact may be studied through the medium of memory, not at the very moment of our perceiving it, but the moment after: and this is really the mode in which our best knowledge of our intellectual acts is generally acquired. We reflect on what we have been doing, when the act is past, but when its impression in the memory is still fresh.

[23]

Ultimately, introspection would be incorporated into certain areas of psychology. Wilhelm Wundt and Edward Titchener would forward the cause of systematic introspection into the early 20^th^ century as they founded *structuralism*, the first paradigm in psychology. Consistent with some of Mill’s ideas (e.g., division of “...the mind into faculties”), structuralists attempted to parse perceptions into essential component parts in order to understand the composition of the mind [25,28]. Later, clinical scientists, informed by the cognitive revolution of the mid-20^th^ century, integrated introspection into “cognitive” therapies. Some of these therapies use mental exercises such as “automatic thought records” to help individuals experiencing distress to identify and modify maladaptive thought patterns.

It must be noted that, although Mill strongly believed in the utility of introspection, he did not explicitly negate the potential utility of examining *outcomes* of cognition. For example, he writes:

We know of our observings and our reasonings, either at the very time, or by memory the moment after; in either case, by direct knowledge, and *not* (like things done by us in a state of somnambulism) *merely* by their results

([23], italics added)

Mill further suggested that an empirical science of mind would require more than just introspection and rational analysis. Although externally valid experimental methods were not in place, he recommended that psychology develop in this direction. He writes:

No mere contemplation of the phenomena, and partition of them by the intellect alone, will of itself accomplish the end we now have in view. Nevertheless, such a mental partition is an indispensable first step…To determine that point (of true cause and effect) we must endeavor to effect a separation of the facts from one another, not in our minds only, but in nature. The mental analysis must take place first.

[1]

Mill’s allusions to “results” and “nature” in the last two passages are consistent with what would become *functionalism*, an approach focused on the processes of thought and their adaptive use for an organism. Functionalist psychologists sought to illuminate the useful effects of cognition, such as learning, through mental testing, psychophysical methods, and experiments [25]. Functionalist ideas evolved, and in the latter part of the 20^th^ century, indirect measurement of cognition became the chosen method of information processing approaches, which examine behavioral reaction time and accuracy in response to standardized cognitive tasks (e.g., discriminating words from non-words). In a sense, structuralism and functionalism share common ancestry with Mill’s ideas. Or, from a different perspective, the passages presented here underscore Mill’s knack for synthesizing (what would later become) competitive paradigms.

#### 3.3.1. Divided Attention and Selective Attention

Whether by introspection or by interpretation of behavior, Mill was an attentive observer of cognition, and he seems to have acknowledged phenomena that are now described as divided attention and selective attention. In modern cognitive science, divided attention is usually distinguished by one’s attempt to pay attention to more than one task at a time, whereas selective attention occurs when one focuses on a stimulus while blocking out irrelevant stimuli.

In the 19^th^ Century, Mill observed that “attention is weakened by being divided” [23]. This concept was adopted and molded by pioneer psychologists near the close of the 20^th^ century. For example, in 1890 William James assumed that conscious attention could only focus on one task at a time, and that any simultaneous activity must be occurring on an automatic, unconscious level [29]. The notion that attentional resources are limited and divisible still proliferates, and it has manifested itself in cognitive laboratory tasks that evoke and detect outcomes of divided attention. To complete these tasks, however, individuals must not only divide their attentional resources; they must focus their attention toward task-relevant stimuli (e.g., Stroop color-word task, dichotic listening task, binocular rivalry task). After all, if attention is finite, then it would be adaptive to allocate it selectively toward information relevant to one’s goals.

In *A System of Logic*, Mill articulates the basis for this type of selective attention, which can be biased from one person to the next. He observed, “One person, from inattention, or attending only in the wrong place, overlooks half of what he sees; another sets down much more than he sees, confounding it with what he imagines, or with what he infers” [1]. 

In this passage, Mill illustrates several essential characteristics of cognition. First, humans can concentrate attention selectively on a single stimulus within a far more complex array of stimuli (*i.e*., attends to half of what one sees). Research in recent decades has indicated that selective attention is executed by simultaneously spotlighting attention on the stimulus of interest and inhibiting attention toward (*i.e.*, blacking out) extraneous information [30,31]. Second, Mill suggests that experience affects which information comes into one’s associative network (e.g., “attending only in the wrong place…”). Third, the associative network may color or distort information as it is perceived (e.g., “sets down much more than he sees, confounding it with what he imagines”). 

The principle that perceptions may inaccurately reflect objective reality presages Adams’ necessary distinction that bona fide cognition must be evaluable and can be false [7]. Mill noted that humans are often unaware of this distinction, as our thoughts fall prey to “mistaking abstractions for realities” [23]. This perceptual principle also seems to be an ancestor of cognitive theories of psychopathology initially disseminated by Aaron Beck [32]. For example cognitive theories of depression propose that negatively biased beliefs, interpretations, attention, and memory can develop from negative experiences and can promote depression. Mill seemed to recognize that the allocation and focus of attention determines what one learns, knows, and experiences.

#### 3.3.2. Meta-cognition

Mill noted that introspection directs attention inward and provides “the knowledge…of what passes in our minds” [23]. It is essentially a form of meta-cognition, or thinking about thinking. More broadly, meta-cognition has been defined as knowledge, beliefs, processes and strategies that observe, evaluate, or direct thinking [33]. Today, there are thousands of peer-reviewed publications about meta-cognition. On the one hand, there is evidence to suggest that meta-cognition can be limited—as Mill conceded—or even problematic [34]. For example, Nisbett and Wilson’s classic review concluded that people often misperceive knowledge of the cognitive processes involved in how they respond to objective stimuli [35]. Additionally, unrealistic meta-cognitive beliefs (e.g., belief that intrusive thoughts will cause catastrophic outcomes) can compromise well-being [33,36].

On the other hand, much of the meta-cognition literature suggests that knowledge of “our observings and our reasonings” can be useful, even if that knowledge can reflect subjective perceptions and causal narratives more than objective inner workings of our cognitive processes [34]. Meta-cognitive ability is an individual difference variable that relates to adaptive social behavior and mental health [37]. Moreover, meta-cognitive awareness is a *skill* fostered in mindfulness-based psychological interventions, often effecting improved cognitive, behavioral, and emotional regulation [38,39]. Thus, introspection can be used as a way to understand and even optimize cognitive functions. 

Mill’s meta-cognition helped him develop his conceptions of cognition and bolstered his belief in introspection as a method. In this way, introspective methods survived through contentious battles in the early development of psychology and may have helped define our modern understanding of cognition. 

## 4. Discussion

Consistent with this Special Issue of *Behavioral Sciences*, John Stuart Mill strived to discover and define what cognition is. This article has attempted to examine Mill’s ideas about mental phenomena and to identify how some of those ideas have been transmitted to a modern understanding of cognition. To this end, the present analysis has considered the cultural ideas Mill acquired and the ideas about cognition that he disseminated. However, before drawing any conclusions about past or current conceptions of cognition, one must consider other matters regarding the evolution and spread of ideas.

When scientific theories break, they remain broken (e.g., phrenology, geocentric model of the universe); however dormant ideas may re-emerge when the environment becomes receptive to them. Sometimes theories are suppressed by a dominant paradigm. For example, Thomas Moore’s *Cognitive Psychology* framework appears to have been inhibited by a prevailing behaviorism [16,28]. Other theoretical frameworks of cognition have recycled over time, with newer iterations possessing a greater level of sophistication [21]. In these cases, the paradigm may be thrust back into prominence by new technology. For example, recent iterations of associationism (connectionism) and neurophysiological consilience with cognition have been revitalized with the help of robust computer modeling and neuroimaging equipment. The paradigm shifts and a new organic period ensues (e.g., “the decade of the brain”).

In Mill’s time, it was unknown whether the theories he espoused would be broken or live on in the marketplace of ideas. His ideas were cogent but controversial. For example, his appeal for a science of mental phenomena defied purely physical or metaphysical explanations, such as the longstanding church doctrine that separated mind from body. As it would turn out, some of Mill’s ideas would do battle for another century. In particular, the Mill-Comte debate presaged themes of important paradigm clashes within psychology [40], such as functionalism *versus* structuralism (preferentially valuing the application or composition of thought), structuralism *versus* behaviorism (debating the empirical basis of introspection), and behaviorism *versus* cognitivism (debating the empirical basis of mental processes). Ultimately, from Mill’s time to now, his vision of a robust, long-lived empirical science of mind has become self-sustaining, albeit modified, because it has produced methods and evidence that continue to support it. At the same time, Mill presaged modern speculation that the psychological study of cognition will become but a chapter in the history of neuroscience [5].

Conclusions of any retrospective analysis must be considered judiciously. Cognition is the tool of investigating and interpreting historical artifacts, and, as Mill alluded, attention, memory, and interpretation functions can be biased. As a result, a retrospective analysis, such as this one, can overlook inconsistencies and may project current mental frameworks onto past texts when those linkages may not be warranted [28]. This can resemble a sort of retroactive interference, whereby the newer memory structure distorts or disrupts access to an older memory structure. Indeed, the present analysis prompted selective searches for Mill passages that resembled ideas expressed in later eras, and these connections may not be as direct as they appear.

A related liability of a retrospective analysis is that human reasoning can fall prey to *post hoc* fallacy, or the problematic conclusion that because one condition preceded another, the former must have caused the latter. Minds that are not linked by time, place, or heredity may nonetheless be predisposed to certain types of thought processes and contents. For example, Ulrich Neisser reported that he knew nothing of Thomas Moore’s 1939 book [16] when he published his own *Cognitive Psychology* text in 1967 [41]. Likewise, it cannot be assumed that every psychologist writing about cognition was (knowingly or unknowingly) influenced by Mill’s relevant 19^th^ Century ideas. Thus, an analysis like the present one can only indirectly infer a contributory relationship between similar ideas expressed by different figures and movements. Despite these limitations, a retrospective analysis can investigate, and at a general level track and explain the trajectory of knowledge and ideas across time.

Since Mill’s era, the science of cognition (see [6,16,41]) has been guided by the principle that a strong theoretical premise must be supported by empirical evidence. Through this methodology the “chaos” Mill described has been decomposed into a “multitude” of experimental findings and an ever-expanding knowledge base. In turn, contemporary descriptions of cognition have come to encompass a wide range of faculties and phenomena, studied by a collection of interested disciplines (e.g., cognitive psychology, cognitive science, neurology, neuropsychology, neuroscience, behavioral science, philosophy of mind, computer science/artificial intelligence). At this point, new discoveries and new questions are outpacing definitional consensus. In response, this article has reviewed some definitional criteria that might help distinguish what qualifies as cognition. In short, cognition is regarded as information processing that originates within a system (e.g., a human), which can be evaluated for correctness, and which can be used in the service of the system. Continued refinement of definitional criteria for cognition may help focus cognitive science. 

Given this review’s focus on the transmission of ideas, this analysis also raises for future examination the question, *who* will define cognition? A general consensus may emerge from grappling with *what* and *where* questions and deciding upon definitional criteria. Or, perhaps different provincial definitions of cognition will evolve and spread to best serve specific disciplines.

## 5. Conclusions

Although it is unclear whether and how conceptions of cognition will change moving forward, it is apparent that John Stuart Mill had a role in shaping current perspectives. In his time, Mill forwarded ideas that would become foundational to the scientific study of cognition. At a fundamental methodological level, Mill’s empirical emphasis on operationalizing antecedents and consequents kept the study of mental phenomena from being lost to ephemeral metaphysics. Regarding theoretical concepts, Mill’s version of associationism transmuted into influential behaviorist concepts and connectionist models of cognition. Mill’s ideas related to introspection, divided attention, and selective attention also live on in modified forms within the basic and applied sciences of cognition. The present analysis concludes that the attributes of Mill and his theory of mental phenomena, along with the receptive sociocultural context of mid-19^th^ century Britain, explain why his ideas have been adopted and applied, and remain relevant in the present era.

## References

[1] Mill J.S., Benjamin L.T. (2009). A system of logic. Excerpt reprinted in A History of Psychology.

[2] (2012). Cognition. Oxford English Dictionary.

[3] Chaney D.W. (2013). An overview of the first use of the terms *cognition* and *behavior*. Behav. Sci..

[4] Walter S., Kästner L. (2012). The where and what of cognition: The untenability of cognitive agnosticism and the limits of the Motley Crew Argument. Cogn. Syst. Res..

[5] Stone T., Davies M., Braisby N., Gellatly A. (2012). Theoretical issues in cognitive psychology. Cognitive Psychology.

[6] Anderson J.R. (2005). Cognitive Psychology and its Implications.

[7] Adams F. (2010). Why we still need a mark of the cognitive. Cogn. Syst. Res..

[8] Damasio A. (1994). Descartes Error: Emotion, Reason, and the Human Brain.

[9] Habibi D.A. (2001). John Stuart Mill and the Ethic of Human Growth.

[10] Capaldi N. (2004). John Stuart Mill: A Biography.

[11] Mill J.S. (1873). Autobiography.

[12] Wilson F., Zalta E.N. (2007). John Stuart Mill. The Stanford Encyclopedia of Philosophy.

[13] Kuhn T.S. (1962). The Structure of Scientific Revolutions.

[14] Comte A., Martineau H. (1855). The Positive Philosophy of Auguste Comte.

[15] Gibson E.J. (1994). Has psychology a future?. Psychol. Sci..

[16] Moore T.V. (1939). Cognitive Psychology.

[17] Mathews A., Mackintosh B. (1998). A cognitive model of selective processing in anxiety. Cogn. Ther. Res..

[18] Damasio A. (1999). The Feeling of What Happens: Body and Emotion in the Making of Consciousness.

[19] Wilson F., Skorupski J.M. (1998). Mill on psychology and the moral sciences. The Cambridge Companion to Mill.

[20] Schmidgen H. (2003). Wundt as chemist? A fresh look at his practice and theory of experimentation. Am. J. Psychol..

[21] Mandler G. (2002). Origins of the cognitive (r)evolution. J. Hist. Behav. Sci..

[22] Barrouillet P., Bernardin S., Portrat S., Vergauwe E., Camos V.  (2007). Time and cognitive load in working memory. J. Exp. Psychol-Learn Mem. Cogn..

[23] Mill J.S. (1865). August Comte and Positivism.

[24] Plunkett K., Juola P. (1999). A connectionist model of English past tense and plural morphology. Cogn. Sci..

[25] Benjamin L.T., Benjamin L.T. (2009). Structuralism and functionalism. A History of Psychology.

[26] Zhang X., Mathews R., Sun R. (2006). Modeling meta-cognition in a cognitive architecture. Cogn. Syst. Res..

[27] Heyd J. (1989). Mill and Comte on psychology. J. Hist. Behav. Sci..

[28] Surprenant A.M., Neath I. (1997). T.V. Moore’s (1939) Cognitive Psychology. Psychon. Bull. Rev..

[29] Spelke E., Hirst W., Neisser U. (1976). Skills of divided attention. Cognition.

[30] Houghton G., Tipper S.P., Dagenbach D., Carr T. (1994). A model of inhibitory mechanisms in selective attention. Inhibitory Mechanisms in Attention, Memory and Language.

[31] Neill W.T., Valdes L.A., Terry K. M., Dempster F.N., Brainerd C.J. (1995). Selective attention and the inhibitory control of cognition. Interference and Inhibition in Cognition.

[32] Beck A.T. (1967). Depression: Causes and Treatment.

[33] Gwilliam P., Wells A., Cartwright-Hatton S. (2004). Does meta–cognition or responsibility predict obsessive-compulsive symptoms: A test of the metacognitive model. Clin. Psychol. Psychother..

[34] Rosenthal D.M. (2000). Consciousness, Content, and metacognitive judgment. Consc. Cogn..

[35] Nisbett R.E., Wilson T.D. (1977). Telling more than we can know: Verbal reports on mental processes. Psychol. Rev..

[36] Wells A. (1995). Meta-Cognition and Worry: A Cognitive Model of Generalized Anxiety Disorder, Behav. Cogn. Psychother..

[37] Reynolds S., Girling E., Coker S., Eastwood L. (2006). The effect of mental health problems on children’s ability to discriminate amongst thoughts, feelings and behaviours. Cogn. Ther. Res..

[38] Eberth J., Sedlmeier P. (2012). The effects of mindfulness meditation: A meta-Analysis. Mindfulness.

[39] Ritschel L.A., Cheavens J.S., Nelson J. (2012). Dialectical behavior therapy in an intensive outpatient program with a mixed-diagnostic sample. J. Clin. Psychol..

[40] Robinson D.N. (1982). Toward a Science of Human Nature.

[41] Neisser U. (1967). Cognitive Psychology.

